# Previremic Identification of Ebola or Marburg Virus Infection Using Integrated Host-Transcriptome and Viral Genome Detection

**DOI:** 10.1128/mBio.01157-20

**Published:** 2020-06-16

**Authors:** Emily Speranza, Ignacio Caballero, Anna N. Honko, Joshua C. Johnson, J. Kyle Bohannon, Lisa Evans DeWald, Dawn M. Gerhardt, Jennifer Sword, Lisa E. Hensley, Richard S. Bennett, John H. Connor

**Affiliations:** a Boston University School of Medicine, Department of Microbiology and National Infectious Diseases Laboratories, Boston, Massachusetts, USA; b Bioinformatics Program, Boston University, Boston, Massachusetts, USA; c Integrated Research Facility, National Institute of Allergy and Infectious Diseases, National Institutes of Health, Frederick, Maryland, USA; St. Jude Children’s Research Hospital

**Keywords:** diagnostic, Ebola virus, filovirus, host response, Marburg virus, presymptomatic, systems biology, transcriptomics

## Abstract

Outbreaks of filoviruses, such as those caused by the Ebola (EBOV) and Marburg (MARV) virus, are difficult to detect and control. The initial clinical symptoms of these diseases are nonspecific and can mimic other endemic pathogens. This makes confident diagnosis based on clinical symptoms alone impossible. Molecular diagnostics for these diseases that rely on the detection of viral RNA in the blood are only effective after significant disease progression. As an approach to identify these infections earlier in the disease course, we tested the effectiveness of viral RNA detection combined with an assessment of sentinel host mRNAs that are upregulated following filovirus infection. RNAseq analysis of EBOV-infected nonhuman primates identified host RNAs that are upregulated at early stages of infection. NanoString probes that recognized these host-response RNAs were combined with probes that recognized viral RNA and were used to classify viral infection both prior to viremia and postviremia. This approach was highly successful at identifying samples from nonhuman primate subjects and correctly distinguished the causative agent in a previremic stage in 10 EBOV and 5 MARV samples. This work suggests that unified host response/viral fingerprint assays can enable diagnosis of disease earlier than testing for viral nucleic acid alone, which could decrease transmission events and increase therapeutic effectiveness.

## Abstract (precis)

Current molecular tests that identify infection with high-consequence viruses such as Ebola virus and Marburg virus are based on the detection of virus material in the blood. These viruses do not undergo significant early replication in the blood and, instead, replicate in organs such as the liver and spleen. Thus, virus begins to accumulate in the blood only after significant replication has already occurred in those organs, making viremia an indicator of infection only after initial stages have become established. Here, we show that a multianalyte assay can correctly identify the infectious agent in nonhuman primates (NHPs) prior to viremia through tracking host infection response transcripts. This illustrates that a single-tube, sample-to-answer format assay could be used to advance the time at which the type of infection can be determined and thereby improve outcomes.

## INTRODUCTION

Diagnostic assay development is an ever-evolving area. Often, the goal is to develop the most sensitive method to detect virus-specific nucleic acids from the infectious agent using nucleic acid amplification technologies such as real-time PCR (RT-PCR) (1–3) or loop-mediated amplification (4, 5). Though these methods have high levels of specificity, the detection of virus material in the blood—the standard approach for diseases such as Ebola virus disease (EVD) and Marburg virus disease (MVD) diagnosis—limits the overall ability of this assay to detect infection, as these viruses do not undergo significant early replication in the blood but, instead, replicate in organs such as the liver and spleen (6). Thus, virus begins to accumulate in the peripheral blood only after significant replication has already occurred in these organs, making viremia an indicator of infection after significant viral propagation has been established.

While disease detection in the symptomatic stage of infection can be sufficient for many diseases, earlier diagnosis of disease is especially desirable for viruses such as Ebola virus (EBOV) and Marburg virus (MARV) to allow early intervention and quarantine. It has been shown that earlier palliative care or postexposure prophylaxis during EBOV infection is associated with improved prognosis (7, 8), linking early detection and diagnosis to better outcomes. Early detection of infected individuals also could have greater potential in helping to control the spread of an outbreak and create better predictions of the potential scope of future outbreaks (9). In the 2013–2016 EBOV outbreak in West Africa, reverse transcription-quantitative PCR (qRT-PCR) for the virus genome had variable ability to determine infection up to 72 h after the onset of symptoms, a critical time in a disease where death can come quickly (10). Together, this argues strongly for developing better diagnostic procedures for diseases such as Ebola virus disease (EVD) and its relative, Marburg virus disease (MVD), that maximize early diagnosis.

One potential approach to promote early diagnosis of filovirus infections is to track the host response to infection. Several studies have shown that the host response can be used to differentiate viral disease from noninfectious inflammatory diseases (11) or other infections, including bacterial and parasitic infections (12–15). The clinical application of identifying host gene expression patterns based on mRNA quantification can differentiate the microorganisms at the center of infections compared to the current diagnostic models (12, 13). Intriguingly, in controlled human and nonhuman primate infections, individual subjects could be successfully identified as infected prior to the onset of symptoms by analyzing changes in host mRNA abundance (16). These findings support the hypothesis that diagnosis of viral infection can be accomplished earlier than currently possible by looking for host responses to viral disease.

The concept of early detection of viral diseases is particularly relevant for infections that are associated with high fatality rates where early diagnosis can lead to better outcomes. Differential regulation of host RNAs in the circulating immune system has been identified in many transcriptomic studies of EBOV infection in nonhuman primates (17–22). In differential-onset models of EBOV disease, host mRNAs signaling future symptomatic infection can appear as many as 4 days prior to fever onset (17). A similar previremic response has been noted as early as 1 to 3 days prior to viremia in nonhuman primate (NHP) models of Lassa virus (LASV) or MARV infection (23). These findings suggest that tracking the host response to filoviral infections could be an effective path toward identifying infection before the appearance of symptoms.

We sought to identify a set of RNAs that could serve as a diagnostic approach for detecting infection by EBOV or MARV that would function in presymptomatic and symptomatic patients. We hypothesized that combining detection of early host responses to infection with virus-specific probes would provide a method capable of early differential diagnosis of Ebola and Marburg virus infection. To test this hypothesis, we established a single-tube assay that combined NanoString probes targeting mRNA present in the peripheral blood that predicted general viral infection, mRNA probes that distinguished EBOV infection from MARV infection, and probes that identified EBOV and MARV genomes. We tested the performance of this approach using blood samples from four independent animal studies with EBOV or MARV infection. Our results suggest that an iterative algorithm to determine viral infection and its causative agent is possible in the context of severe emerging viral diseases.

## RESULTS

### Identification of conserved innate immune response genes following EBOV infection.

Our first goal was to identify the most robustly expressed early responses to EBOV infection. Several reports have identified rapidly upregulated mRNAs in blood and peripheral blood mononuclear cells (PBMCs) in response to EBOV infection (17–21). We sought mRNAs that were consistently upregulated at early time points postexposure in existing data sets of NHPs infected with EBOV Kikwit (19), EBOV Makona C07 (20), EBOV Makona SL 3864.1 (17), or EBOV Makona C05 (24, 25). These studies represented various strains and isolates, routes of exposure, and variations in whether PBMCs or whole blood was used for sequencing.

**FIG 1:**
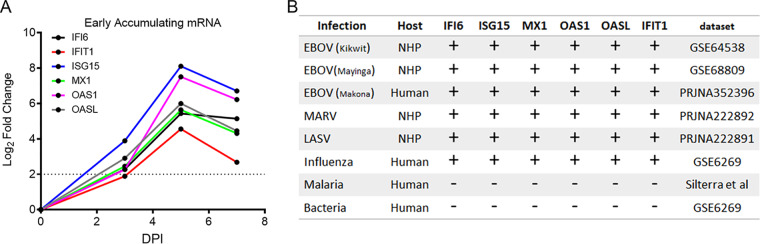
Conserved interferon-stimulated genes showing early and sustained expression following virus infection. (A) Next-generation sequencing (NGS)-determined changes in mRNA abundance for six interferon-stimulated genes at increasing times postinfection (DPI, days postinfection). The *y* axis represents the log_2_ fold change for the 12 animals from preinfection (0DPI) to the DPI for IFI6 (blue) IFIT1 (red), ISG15 (blue), MX1 (green), OAS1 (magenta), and OASL (gray). (B) Table representing comparative-expression results for each of the mRNAs shown in panel A in nonhuman primate (NHP) models of infection of EBOV, MARV, and LASV and human cases of EBOV, influenza, malaria, and Haemophilus influenzae (influenza). +, Upregulation of the gene compared to uninfected controls; –, negligible upregulation of the gene compared to uninfected controls.

Across the multiple studies used for discovery of early gene markers, we found that certain interferon-stimulated genes (ISGs) were consistently upregulated in all symptomatic animals in all data sets. As an example, the ISGs IFI6, IFIT1, ISG15, MX1, OAS1, and OASL showed strong upregulation (log fold change, >1.5, false-discovery rate [FDR], <0.05) across all data sets analyzed. This is illustrated in Fig. 1A, which shows the average change in expression in one cohort of NHPs following EBOV Mak C05 challenge at days 3, 5, and 7 postinfection. In this cohort of 12 animals, all 6 mRNAs showed accumulation in the blood at day 5 postinfection, and all but IFIT1 showed high expression at day 3 postinfection (log fold change, >2). These genes have also been observed to be upregulated in humans and NHPs infected with other Ebola virus isolates (18–20, 22), hemorrhagic fever viruses (23), and viruses such as influenza (12, 16, 26). These mRNAs did not show significant accumulation in response to bacterial (26) or parasite (27) infection (Fig. 1B).

### Development of a virus- and host-targeted NanoString code set to identify early stages of infection.

**FIG 2:**
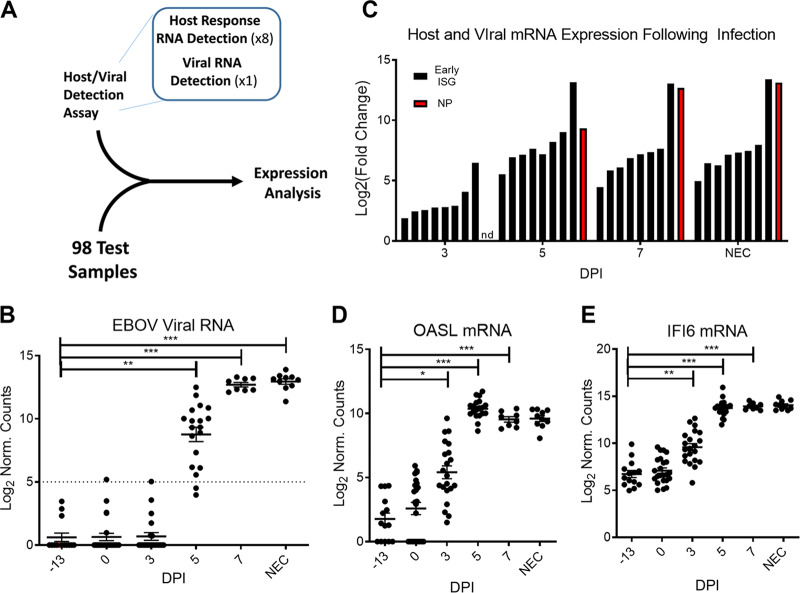
Detection of virus RNA and host-response RNA present in the blood of infected NHPs after EBOV infection using NanoString. (A) Schematic representation of the experiment. (B) Number of viral genome copies detected in each sample tested. Each point represents a data sample from an individual NHP at that time, and the dotted line represents the threshold of significance above background for the probe. (C) Bar graph representing the average fold change of each host mRNA (black) and of the viral genome (red) in the NanoString assay. Each bar represents the average increase from baseline for each NanoString probe at each day postinfection. (D and E) Expanded graphs illustrating the variance and level of mRNA abundance for host response genes ISG15 (D) and IFI6 (E). Each point represents a sample from an individual NHP at that time. The lines represent the average values, and error bars represent the SEM. *, *P* < 0.05; **, *P* < 0.001; ***, *P* < 0.0001.

**FIG 3:**
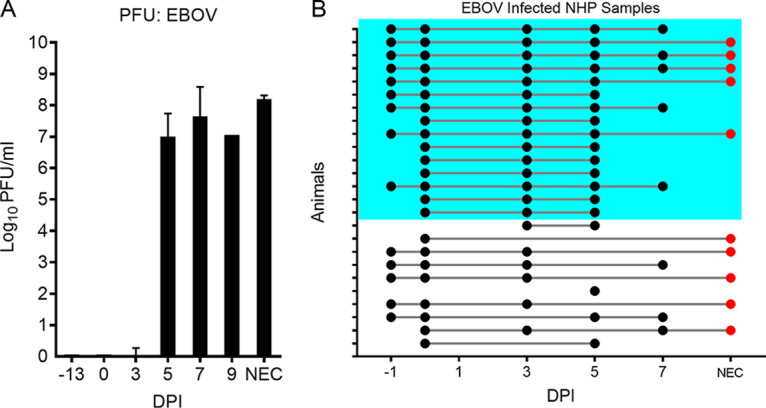
Appearance of viremia and sample collection data supporting initial testing host-based early infection assay. (A) Summary of viremia information for animals used in testing. Graph shows viremia (PFU/ml) prior to infection and at increasing times postinfection. (B) Summary of the whole-blood RNA samples collected for analysis with NanoString. The different days postinfection are shown on the *x* axis, with –1DPI and 0DPI being from preinfection samples. Points connected by a line represent serial sampling from the same animal. Red points are samples taken after necropsy. The samples in the blue box represent the subset of animals for which sampling is available at preinfection, early infection (3DPI), and late infection (5DPI).

To test the potential for early-responsive host mRNAs to serve as markers of early EBOV infection, we created a NanoString multiplex probe set (28, 29). We incorporated nine RNA probes into our initial assay, one probe to recognize the EBOV genome within the nucleoprotein (NP) gene sequence and eight probes to recognize ISG host transcripts associated with early host response to viral infection (host/viral detection assay V1; see Fig. S1 in the supplemental material for the gene list). We hypothesized that an early host signature of infection would be detectable prior to viremia using our NanoString code set. We tested this hypothesis using 98 samples from 23 EBOV-challenged NHPs (Fig. 2A). The time points analyzed included preinfection (2 time points for each NHP), day 3 postinfection (3DPI, presymptomatic), day 5 postinfection (5DPI, symptomatic), day 7 postinfection (7DPI, symptomatic), and upon necropsy (NEC). In this study, most animals first showed viremia by day 5 postinfection by plaque assay (Fig. 3).

10.1128/mBio.01157-20.1 FIG S1 Design and testing strategy for RNA-based infection-detecting multiplexed assays. From left to right, the diagram shows the initial assay components included in the V1 assay, with host RNAs detected shown in black and viral RNA shown in blue. Moving right, the diagram shows that following testing, new mRNA-detecting probes were added to create a V2 assay that was capable of predicting disease stage. mRNA probes added at this stage are shown in green. The final iteration involved the addition of a probe to detect MARV genomic RNA as well as additional host mRNA-detecting probes (purple) to lead to a third-generation assay, the EBOV/MARV discrimination assay. Download FIG S1, DOCX file, 0.03 MB. Copyright © 2020 Speranza et al. 2020 Speranza et al. This content is distributed under the terms of the Creative Commons Attribution 4.0 International license.

The EBOV-specific probe showed no significant levels above background of the EBOV genome at day 0 or 3DPI in our assay, which is consistent with the negligible levels of viremia seen by plaque assay. By 5DPI, there were significant levels of EBOV genome for most NHPs. By 7DPI, all NHPs showed detectable levels of EBOV genome (Fig. 2B). In contrast, the host mRNA response to infection was more readily detectable than viral RNA accumulation in these samples at 3DPI. All eight early viral infection probes showed increased accumulation of host response mRNAs at 3DPI (Fig. 2C).

Accumulation of host mRNAs increased by 5DPI, and expression was maintained through 7DPI and at necropsy. Analysis of expression levels for two of these probes—OASL and IFI6—showed various levels of basal gene expression (Fig. 2D and E) with significant but highly variable expression by 3DPI (*P* < 0.05). By 5DPI, these genes had reached maximum expression levels with very little variation in expression between animals. This high level of expression was maintained throughout the remaining disease course.

To rigorously test the hypothesis that host mRNA accumulation could be detected prior to viral RNA appearance in the blood, we examined viral and host RNA accumulation in the 15 NHPs for which we had data from preinfection, 3DPI, and 5DPI samples (Fig. 3B, blue box). Defining the host response as at least two host mRNAs expressed 4-fold above baseline in an individual animal and viremia as 3 counts above the preinfection level, all NHPs showed a spike in host mRNA accumulation at 3DPI. A total of 14 of the animals did not show viral RNA increases at 3DPI, and one showed viremia coincident with host gene expression (Table 1, animals AA to NN). This illustrated that NanoString quantification of mRNAs in the blood of infected NHPs identified a host infection signal prior to viral RNA accumulation and that viral RNA detection paralleled the appearance of infectious virus in the blood (compare Fig. 2B and Fig. 3A).

**TABLE 1:** Timing of host mRNA signature and viral signatures of Ebola virus infection

Animal^*a*^	ISG+^*b*^ (DPI)	EBOV+^*c*^ (DPI)	Early?^*d*^
AA	3	5	Yes
BB	3	5	Yes
CC	3	5	Yes
DD	3	5	Yes
EE	3	5	Yes
FF	3	5	Yes
GG	3	5	Yes
HH	3	5	Yes
II	3	5	Yes
JJ	3	5	Yes
KK	3	5	Yes
LL	3	5	Yes
MM	3	5	Yes
NN	3	5	Yes
OO	3	3	No

a Anonymized NHP IDs.

b ISG+, the day at which at least 2 host mRNAs achieved significance over background in NanoString assays.

c EBOV+, the day when the EBOV NP gene was detectable in NanoString assays.

d Early indicates whether the host response signature was detected prior to EBOV RNA.

### Development of a baseline-independent method for early identification of EBOV.

We next tested whether we could *a priori* identify and predict the stage of virus infection. This was done to examine whether classification could be effectively carried out in the absence of a preinfection or baseline sample. To develop such a classification approach, we first expanded our NanoString testing panel. We included mRNAs from the viral/host detection assay V1 as a means of separating uninfected from infected samples. Eight additional mRNAs (CRYL1, PPAP2B, RRAD, S100A12, STAT2, STK38L, TGFB1, and CD3D) were incorporated as part of viral/host detection assay V2. This probe set was used to interrogate the large 98-sample test set described in Fig. 3B. The results from this test indicated different patterns of accumulation for each host mRNA over the course of disease.

**FIG 4:**
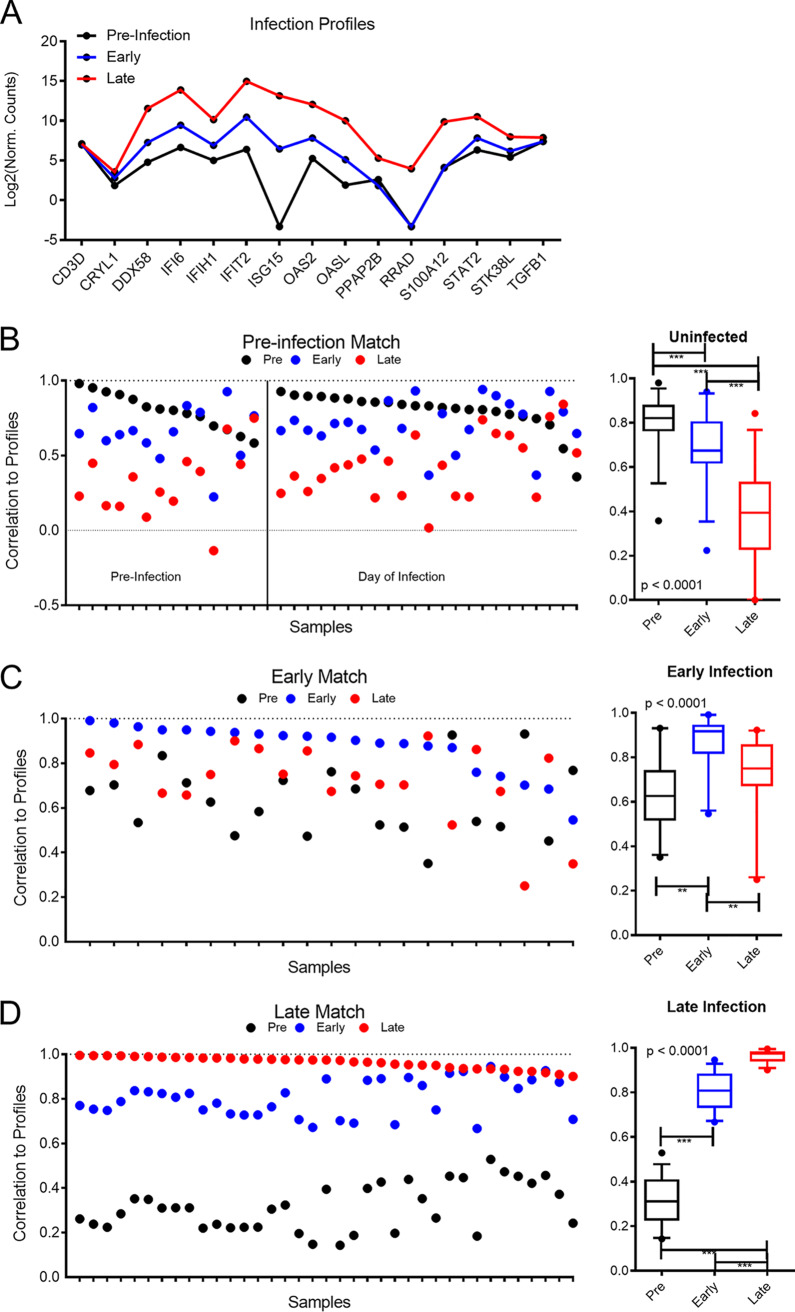
Gene expression profile approach for classifying and staging virus infection. (A) Gene expression profile of 15 host genes at different times postinfection. The median gene expression values were determined from all preinfection (–13DPI and 0DPI) samples, all early (3DPI) samples, and all late (5DPI) samples to generate profiles of gene expression. Preinfection median points are contained on the black line, early points are in blue, and late points are in red. (B to D) Computed profile correlation of each sample tested to preinfection, early infection, or late infection gene expression profile using leave-one-out validation. The *y* axis represents the calculated level of correlation for each sample tested, with perfect correlation equal to 1. Samples are colored by preinfection (black), early infection (blue), and late infection (red). The quartile plots on the far right of each panel, B to D, represent the distribution of correlations to each profile for all samples. **, *P* < 0.001; ***, *P* < 0.0001.

We created expression profiles for the different times postinfection using the median value of expression for each gene across the samples within a given group. When these values were plotted for each RNA probe, they generated differential patterns of RNA accumulation for the different stages of disease (Fig. 4A). We used these differential patterns of accumulation to create a profile-based method to classify samples into three categories, uninfected, early/previremic, and late infection.

We first examined the 37 samples that represented NHPs prior to infection (–13DPI and 0DPI). When the RNA expression levels for each of these samples were compared to uninfected, early, and late infection profiles using a leave-one-out cross-validation, 23 of these 37 samples correlated most strongly with the preinfection profile (Fig. 4B). Of the 13 samples that did not match the preinfection profile best, 12 matched the early infection profile, and one matched the late infection profile. Both preinfection and day of infection had similar false classification rates (28% and 39%, respectively; *P* = 0.5), suggesting that misclassification was not likely a stress response on the day of infection. For the preinfection samples, the correlation values to the different profiles (preinfection to preinfection, preinfection to early infection, or preinfection to late infection) were significantly different from each other (*P* < 0.0001). Additionally, the preinfection correlation values to the preinfection profiles were significantly higher than preinfection to early infection profile correlations and preinfection to late infection profile correlations (*P* < 0.0001 for both) (Fig. 4B).

In samples from animals at the previremic time point (3DPI), 18 of the 21 samples matched best, with either the early profile or late profile having the highest match (Fig. 4C), and 3 most closely correlated with the uninfected samples. Again, the correlation values of early infection samples to early infection profiles was significantly higher than those for early infection samples to the preinfection profile or early infection samples to the late-infection profile. For the late-infection profiles, all the samples were correctly identified as infected; 35 of the 37 samples matched the late profile, and two matched the early profile (Fig. 4D). Together, these data show that when using the profile match method, the samples trend toward matching the late infection profile as disease progresses, yet samples were identified as infected as early as day 3 postinfection. Additionally, for all stages of disease, the average correlation of the samples to the correct profile was significantly higher than that to incorrect profiles (*P* < 0.001 for all) These results also emphasize that while an infection classifier that predicts infection based on host mRNAs alone is capable of identifying infected samples prior to viremia, it can incorrectly classify uninfected samples as being at early stages of infection.

### Testing of general infection classifier on non-EBOV samples.

We next investigated whether we could use this assay to stratify infection in NHPs exposed to MARV, a hemorrhagic fever virus in the same virus family as EBOV. Blood samples from NHPs infected with MARV were obtained from individual animals at preinfection, 3DPI, 6DPI, and upon necropsy (Fig. S2A; 30). Following the preparation of total whole-blood RNA from these samples, we analyzed RNA expression levels using our 15-RNA code set and correlated the results to pre-, early-, and late-infection profiles. Five of the six preinfection samples matched the preinfection profile, and the remaining sample matched the early profile. When we looked at samples from 3DPI, all 5 samples tested were correctly classified as early infection. All 6DPI samples were correctly identified as infected. Early infection identification with the NanoString assay preceded viremia by 3 days (Fig. S2B). These data suggest that the previremic classifier is effective at identifying infection at early times in multiple examples of viral hemorrhagic fever.

10.1128/mBio.01157-20.2 FIG S2 Previremic identification of infection in MARV-exposed NHPs using a NanoString assay. (A) Blood samples from the MARV-exposed NHPs used in this study. Each black dot represents one blood sample taken at the indicated time postinfection. (B) Compressed analysis of sample correlation to expression profiles determined on the EBOV samples. NanoString RNA level results from each sample (*x* axis tic mark) were compared to preinfection, early infection, and late infection RNA accumulation profiles, and the extent of correlation to pre- (black), early (blue), and late (red) infection profiles is shown. In this analysis, perfect correlation equals 1. The highest correlation value would determine if the sample is classified as infected or uninfected, with early and late infection both representing infected. Download FIG S2, DOCX file, 0.1 MB. Copyright © 2020 Speranza et al. 2020 Speranza et al. This content is distributed under the terms of the Creative Commons Attribution 4.0 International license.

### Generation of a virus-specific marker using a combined virus-host classifier.

Based on the ability of these assays to predict and stage infection in blood samples using host RNAs, we also sought to identify host RNAs that showed expression changes following EBOV infections but did not show expression changes in other hemorrhagic fever virus infections, such as the closely related MARV, the rodent-transmitted LASV, or the mosquito transmitted yellow fever (YF). LASV belongs to a different virus family but is known to cause disease in the same region as EBOV (31). To do this, we analyzed RNA-Seq data sets from EBOV, MARV, and LASV infections in NHPs (19, 23) and a microarray data set from YF infections (32). RNAs were selected that showed the greatest difference in expression at early times postinfection in EBOV-infected NHPs compared to the MARV- and LASV-infected NHPs (Fig. S3A). This identified 4 host RNAs (ADAM28, STK38L, ZFYVE1, and MMP8) that were highly upregulated at early times in EBOV infection but not in MARV or LASV infection and 4 host RNAs (TCRA, SIT1, FCER1A, and CD5) that were greatly downregulated in EBOV infection but not in MARV or LASV infection. Additionally, we showed that at day 3 postinfection, there was a unique signature between EBOV (shown by STK38L), MARV (shown by RRAD), and YF (shown by Serpinb9 and Ifit2), suggesting that changes in infections with similar presentations can have unique signatures present in the blood (Fig. S3B).

10.1128/mBio.01157-20.3 FIG S3 Host RNAs that show differential early expression in various viral infections. (A) Specific markers for EBOV infections compared to MARV and LASV. Each graph depicts the normalized log-fold change of one host mRNA at increasing times postinfection to EBOV (black), MARV (red), or LASV (blue). Analysis was done using data from GS64538, PRJNA222892, and PRJNA222891. The top 4 graphs represent mRNAs that showed selective upregulation in EBOV but not MARV or LASV at early times postinfection. The bottom graphs represent mRNAs that showed selective downregulation following EBOV infection at early times postinfection. (B) Selected genes showing unique gene expression at day 3 postinfection compared to controls in Yellow Fever (YF), MARV, LASV, and EBOV. YF data is taken from GSE51972. Data represents log FC compared to uninfected controls. Download FIG S3, DOCX file, 0.1 MB. Copyright © 2020 Speranza et al. 2020 Speranza et al. This content is distributed under the terms of the Creative Commons Attribution 4.0 International license.

The probes distinguishing EBOV from MARV were added to the mRNAs used in host/viral detection assay V2 to create a third-generation NanoString assay that contained a probe for the EBOV genome, a probe for the MARV genome, and various virus-specific host response RNAs (EBOV/MARV discrimination assay; see Fig. S1). We used this NanoString assay to classify 5 animals from MARV-infected NHPs and 14 animals from EBOV-infected NHPs at preinfection, 3DPI, and 5DPI/6DPI time points. The preinfection profiles generated for all infected animals had high correlations with each other (Pearson *R* = 0.98, *P* < 0.0001), suggesting that the animals were indistinguishable prior to infection in our assay. The early EBOV versus MARV profiles had lower correlations with each other (Pearson *R* = 0.77, *P* < 0.0001), and the late profiles for the different infections had very low correlations with each other (Pearson *R* = 0.12, *P* = 0.73).

**FIG 5:**
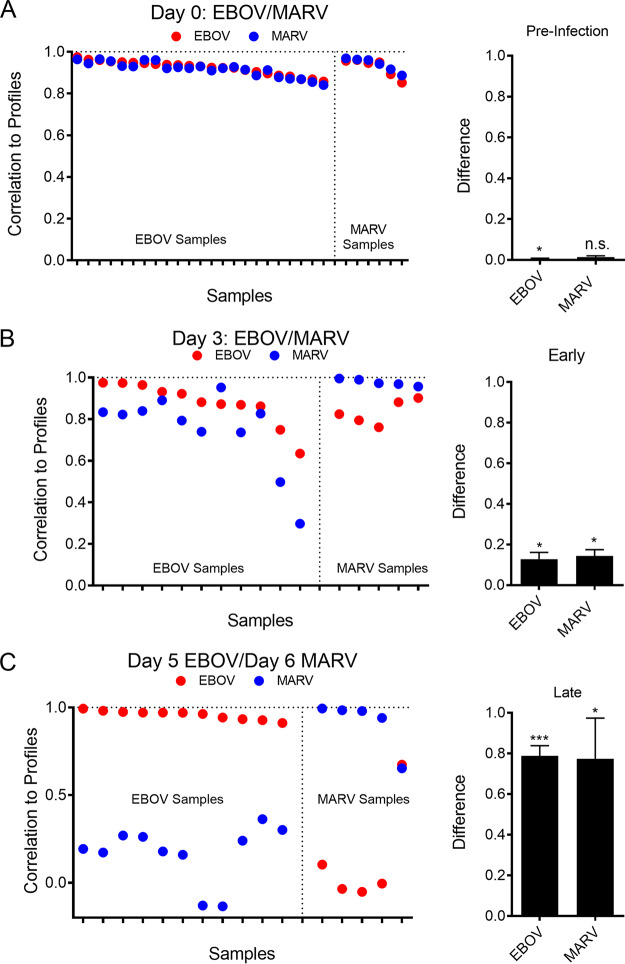
Development of a virus-differentiating classifier using host and virus-specific probes. (A) Correlation to a virus-specific RNA expression profile for individual samples from either EBOV- or MARV-infected NHPs at preinfection. Results shown in this plot represent results obtained from uninfected blood. Red points represent correlation to profile for EBOV; blue points represent correlation to profile for MARV. The bar plot to the far right shows the average difference of the expected profile to the other profile (EBOV sample to EBOV profile – EBOV sample to MARV profile). (B) Correlation of NanoString assay results from day 3 samples to infection profiles for EBOV (red) or MARV (blue) infection. Samples showing a higher correlation to the EBOV than to the MARV profile represent model-identified EBOV infection, while samples with a higher correlation to the MARV profile are called MARV infection. (C) The correlation of NanoString assay results from 5DPI samples (EBOV) or 6DPI samples (MARV) to virus-specific profiles. The bar plot highlights the increased difference in average correlation of the expected profile of infection to the other profile of infection.

We tested how each sample correlated with the different infection profiles using leave-one-out cross-validation. Uninfected samples matched very closely the uninfected sample profile for both EBOV- and MARV-infected NHPs (Fig. 5A). This was expected since the gene expression changes at preinfection should be the same independent of the infectious agent. The difference from the expected profile (e.g., EBOV sample to EBOV profile) to the other profile (e.g., EBOV sample to MARV profile) was minimal (Fig. 5A, far right).

Similar analysis was done comparing samples from 3DPI from both EBOV- and MARV-infected NHPs (Fig. 5B). All but one EBOV sample was correctly identified as EBOV-infected, and all MARV samples were correctly identified. All were negative for viral RNA in the NanoString assay, and virus infection classification at this time was done solely through changes in host-responsive mRNAs. Using just the virus probe as a classifier, neither the EBOV sample nor the MARV sample showed expression of the virus above background at 3DPI (Fig. S4) and did not identify infection at this time point. The difference between the correlation to the expected prediction and other prediction was significantly greater than zero for EBOV (*P* = 0.0031) and MARV (*P* = 0.0095; Fig. 5B, far right).

10.1128/mBio.01157-20.4 FIG S4 Appearance of EBOV and MARV genome RNA in blood samples from EBOV- and MARV-infected NHPs using NanoString. (A) Normalized probe count values for an EBOV genome-specific RNA probe. Each point represents normalized counts from an individual blood sample on the indicated day postinfection. The horizontal line indicates the average value, with error bars signifying the standard deviation. The dashed line represents the background threshold for the probe. Samples from EBOV-infected NHPs are shown in black, and samples from MARV-infected NHPs are shown in red. (B) Similar graph to that in panel A but showing results from the MARV-genome-specific RNA probe. Arrow shows the early infection timepoint (3DPI). Download FIG S4, DOCX file, 0.1 MB. Copyright © 2020 Speranza et al. 2020 Speranza et al. This content is distributed under the terms of the Creative Commons Attribution 4.0 International license.

NanoString results comparing samples from late infection time points (5/6DPI) showed high degrees of association with appropriate late-infection profiles for each infection. All 11 EBOV samples correlated with the late EBOV profile. Four of five MARV samples correlated with MARV late profiles. One MARV sample was incorrectly identified as EBOV due to low levels of MARV-specific RNA. Since at this time point the training of the classifier was driven by the presence of viral RNA, the lack of viral RNA in the one sample drove the incorrect classification. Further testing of additional samples could help tune the model better to identify this type of sample. The difference in the correlation values of the expected profile to the other profile was significant for the EBOV samples (*P* < 0.0001) and for the MARV samples (*P* = 0.018) (Fig. 5C).

## DISCUSSION

This work builds on earlier data showing that there are previremic and presymptomatic markers of infection following exposure to high-consequence viruses that are associated with hemorrhagic fever (17, 18, 20, 23, 24). Here, we developed sample-to-answer assays that determine RNA levels in blood using general markers of infection as well as specific markers of EBOV or MARV infection. Our testing shows that these assays can identify infection prior to the appearance of viral RNA in the blood and can differentiate infection with closely related viruses.

This approach is a step forward for monitoring high-risk individuals for signs of infection and predicting likely infection sources before the onset of symptoms or viremia. Disease modeling approaches have determined that presymptomatic detection is necessary for limiting spread and increasing survival during outbreaks (7, 9), and our results show that tracking both general and specific circulating host responses can help provide this information. Though this study focused on samples collected from NHP models of disease and not human samples, transcriptomic analysis of the human response to EVD has shown that there is a high correlation between the NHP and human responses (33, 34), making our results likely to be translatable.

**FIG 6:**
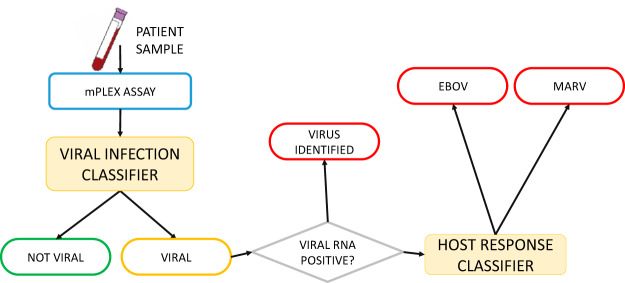
Two-stage host/viral RNA infection classifier. This flowchart depicts an anticipated analysis workflow following the host/virus detection assay probing of an unknown sample. The first step of analysis uses host mRNAs in a general virus infection classifier to determine if the individual is likely uninfected or positive for virus infection. If the sample is positive for infection, the algorithm then looks for the presence of virus RNA. If the sample has ample viral RNA present, then the infection is definitively classified, and the algorithm stops. If viral RNA is not present, then the virus-specific classifier is run. From this, the causative agent is inferred to be EBOV or MARV using host-mRNA abundance.

Our use of a multiplexed host response tracker supports a multipronged infection identification algorithm that will classify presymptomatic samples and determine if they likely came from an EBOV or MARV. An algorithm describing the use of this type of classifier is depicted in Fig. 6. This figure imagines the analysis of an unidentified blood sample using the EBOV/MARV detection assay (Fig. 5; Fig. S1). RNA abundance levels are first run through the “general viral infection” classifier that detects early innate immune responses. This analysis predicts if the sample came from an uninfected individual or from an individual who was likely infected with a virus.

If the sample is positive for markers associated with viral infection, the algorithm moves to a second level that uses mRNA from the other probes in a combined analysis of host mRNA and viral genomes to suggest the potential infectious agent. This represents a hybrid approach that combines looking at the host response while at the same time searching for unique viral products within the same assay system. If the sample comes from a viremic individual, detection of viral RNA drives the algorithm decision. If the sample is not viremic and the infectious agent cannot be identified, the host gene expression pattern is used to infer the causative agent.

We see this two-component approach of following both host response gene signatures and unique viral gene signatures as an important point of investigation for improving the quality and applicability of diagnostics for high-consequence pathogens. During the EBOV outbreak in West Africa in 2013 to 2016 and again in the ongoing North Kivu outbreak in the Democratic Republic of the Congo, health care workers and primary contacts of symptomatic individuals were at significant risk of acquiring the disease. Regular sampling of individuals could provide the opportunity to correctly identify Ebola infection at or prior to onset of fever and could speed treatment of infected individuals in this high-risk pool.

Though this study did not directly assess whether the first step of our infection classifier would effectively flag infection with other viruses, RNAs that we utilize in the viral infection classifier have previously been incorporated as a component of other host-based diagnostic assays that seek to differentiate bacterial from viral infections (11–13). These studies and others (16, 27) found that ISGs such as OAS, DDX58, and MX1 are differentially regulated early in viral infection. In these classifiers, the innate immune mRNAs act to differentiate viral infections against other infectious agents, such as bacteria, parasites, or other causes of inflammatory disease in humans. This gives us confidence that the general infection classifier will not only perform across a broad range of viral infections, but also would distinguish viral infections from parasitic and bacterial diseases.

Our enthusiasm about the usefulness of a host/viral classifier system is tempered by the recognition that additional work remains to be done to further define the strengths and weaknesses of this approach. The current approach would benefit from testing on a larger validation data set, as all testing and training were performed internally. Other early detection probes have been suggested (though not tested) for EBOV and may add additional value in prediction. The requirement for high containment to perform these studies as well as the limited numbers of studies makes this a significant challenge. As more studies are performed, better validation of the general infection classifier and the EBOV-specific gene set can be undertaken. Also, we have currently not taken into consideration coinfections, which are possible and common in the demographic regions where EBOV and MARV cause human outbreaks and have an undefined impact on our genes of interest.

This work highlights that presymptomatic EBOV diagnostics are possible using a few host response genes, building on repeated findings that these genes are expressed at early times postinfection in animal models of disease. As a developed assay, the code sets described here can be used in various scenarios—as an early indicator of appearance of infection in models of filovirus disease that show variable onset (17), as a trigger for treatment for therapeutic intervention, and as a monitoring approach for high-risk individuals involved in the care of EBOV- or MARV-infected individuals.

## MATERIALS AND METHODS

### Infections with EBOV Makona.

The CO5 isolate of Ebola virus isolate Makona (full designation, Ebola virus/H.sapiens-tc/GIN/2014/Makona-CO5; abbreviated name, EBOV/Mak-CO5; GenBank accession number KP096420.1; BioSample number SAMN03611815) was generously provided by G. Kobinger of Public Health Agency Canada and propagated as previously described (lot number IRF0137) (35). Animals were challenged intramuscularly with a target dose of 1,000 PFU. Animals were observed daily for clinical signs of infection and humanely euthanized when they met preestablished euthanasia parameters allowing morbidity as a surrogate for lethality. An extended description can be found in reference 24.

### Infection with MARV.

The Angola isolate of Marburg virus (full designation, Marburg virus/H.sapiens-tc/AGO/2005/Ang-1379v; abbreviated name, MARV/Ang) was used for infections. This stock (lot number IRF0202) was isolated from a fatal human case collected by the CDC and was propagated in VERO C1008 (E6) cells. Cells were obtained from working Cell Bank, NR-596 were obtained through BEI Resources (National Institute of Allergy and Infectious Diseases [NIAID], National Institutes of Health [NIH], Manassas, VA); minimum essential medium-alpha, GlutaMAX, with no nucleosides (Gibco, Thermo Fisher Scientific) supplemented with 2% U.S.-origin, certified, heat-inactivated fetal bovine serum (HI-FBS; Gibco, Thermo Fisher Scientific) was also used. This virus stock has a passage history of VERO E6 passage 4. Following harvest, HI-FBS was QS’d to 10% final concentration prior to cryopreservation. Animals were challenged intramuscularly with a dose of 1,000 PFU. Animals were observed daily for clinical signs of infection and humanely euthanized when they met preestablished euthanasia parameters allowing morbidity as a surrogate for lethality. A more detailed description of the animal model can be found in reference 30.

### RNA extractions and quality control.

Whole-blood samples from infected macaques (either Ebola or Marburg) were combined with 3 volumes of TRIzol LS reagent. Samples were mixed thoroughly and left to incubate for 10 min to inactivate virus. Samples were then transferred to a new tube and sterilized by soaking the tubes in microchem for a minimum of 10 min.

RNA was extracted using the standard TRIzol protocol. First, 200 μl of chloroform was added per 1 μl of sample and shaken by hand. Phase separation was done for 15 min at 12,000 × *g*, and the aqueous layer was saved. To further clean up samples, a second chloroform addition and phase separation step was performed. After the second aqueous phase was placed in a fresh tube, an equal volume of isopropanol was added to the samples, and they were left to incubate at 4°C for 3 h. RNA was then precipitated by spinning the samples at 12,000 × *g* for 10 min, and the supernatant was removed. Then, 75% ethanol was added to the pellet to wash the RNA, vortexed, and spun for 5 min at 7,600 × *g*. The ethanol was removed, and 30 μl of RNase-free water was added. RNA quality and concentration were determined by bioanalyzer analysis using an Agilent bioanalyzer 2100 and RNA Nano 6000 chips.

### NanoString analysis.

Our procedures are similar to those described in other recent EBOV host response analysis approaches (17, 36). A NanoString Elements code set was developed to target 12, 36, or 48 genes. Oligos were generated through Integrated DNA Technologies (IDT). Then, 100 ng of RNA in a maximum 13-μl volume was added to the NanoString reaction. Samples were left to incubate at 37°C for 12 h and held at 4°C until processing. Samples were prepped on a NanoString Max prep station. After sample prep, cartridges were kept at 4°C until reading. Read counts were generated on the NanoString Max digital analyzer station.

### NanoString data normalization.

Raw count files (.RCC) were read into the nSolver Advanced Analysis software 3.0 to perform quality control checks and generate count tables. Quality control included filtering for too few field-of-view (FOV) counts (expected, 270), filtering for binding densities outside the expected range (0.5 to 2.5), checking for positive-control linearity to spike-in RNA, and determining if the lowest positive control (5 pm) was at least greater than 2 s.d. above the mean of the background. Count files were then read into R. Normalization of the counts was performed as follows. First, the positive-control normalization was performed to account for variance across lanes. The geometric mean of the spike-in positive controls for each lane was determined. Then, an average value for these geometric means was calculated. A positive-control normalization value was calculated for each lane by dividing the average across the lanes by the geometric mean for each lane. This was then used as a multiplier for the gene counts in the lanes. Next, normalization for the input amount of RNA for the different samples was determined. A housekeeping gene, RPL37A, was included in the code set. RPL37 shows low variance and high count values across different EBOV infections using HoTResDB (37). For the normalization, a similar process was used as with the positive controls. A mean of the counts across the lanes was determined for RPL37A, and a scaling factor was calculated by dividing the mean across the lanes by the counts of RPL37A within the sample. Then, the positive-control normalized counts were multiplied by this value to account for input control.

### RNA-Seq data processing.

Raw sequencing reads were demultiplexed using the Illumina BaseSpace application. Demultiplexed reads were then downloaded and processed as follows. Filtering of reads for poor quality of the ends of reads, read length, and poor-quality reads was done using the FASTX-Toolkit. After filtering, reads were mapped to the rhesus macaque genome (38) using TopHat 2.1 (39), which calls the aligner Bowtie 2 (40). After mapping of the reads was completed, count tables were generated with HTSeq count (41).

Raw count files were read into R. Reads were normalized using the DESeq2 (41) rlog function, and principal-component analysis was carried out to determine significant outliers. Finally, differential gene expression analysis was performed in DESeq2 to determine significantly differentially expressed genes using standard cutoffs (absolute log fold change, >1; adjusted *P* value, <0.05).

### YF microarray data processing.

Microarray data were acquired from GEO GSE51972. Data were downloaded and normalized in R using Biobase and GEOQuery. Spots with a significant change at day 3 postinfection compared to day 0 for YF infections were calculated using Limma. These were compared to the fold changes from the RNA-Seq data described above for EBOV, LASV, and MARV infections, and genes showing a different pattern of expression were highlighted.

### General infection classification of samples.

Two different approaches were taken to identify general infection using the NanoString code set. The first method was dependent on the preinfection samples for each animal. A fold cutoff of 4 for a minimum of 2 selected genes determined if a sample was positive for infection or identified a change in the EBOV genome 3 counts above the preinfection maximum levels.

The second method used in NanoString was profile correlation. To determine which genes from a larger NanoString code set would still separate samples, a filtering step was first performed. Genes that did not show strong expression (log mean of normalized counts > 0) were removed so that only genes that were strongly expressed were included. A substitution method was used to determine genes that create an expression profile that separates the different days from each other. This method begins with a randomly selected gene set. Then, the profiles are generated using the median counts of the samples within a given group for each gene. The profiles are then correlated with each other to determine how similar or different they are. Then, genes are iteratively substituted into the model to determine if they create lower correlation values. This is run until convergence is reached. This was then run 100 times for a starting number of genes between 5 and 20. An optimal set was chosen based on end correlation values of 15 genes. To perform leave-one-out cross-validation using these selected genes, one sample at a time was removed. Next, the profiles for the different categories were generated using the 15 genes and the remaining samples’ median expression values. Then, the correlation of the left-out sample to the generated profiles was determined, and the highest match was used to categorize the sample. A similar process was done for the virus-specific classifier.

### Statistical methods.

To determine if the correlations of the profiles were different from each other in the general-infection classifier, a Kruskal-Wallis test was performed across the three groups. To determine if one group was significantly different from another, a Mann-Whitney test was performed on the two groups. For the virus-specific classifiers, a standard *t* test was performed to determine if the difference between the expected profile and the other profile was significantly different from zero. Statistical calculations were carried out in GraphPad Prism 5 or in R.
