# Supplementary material for: Previremic Identification of Ebola or Marburg Virus Infection Using Integrated Host-Transcriptome and Viral Genome Detection
Source: mBio. 2020 Jun 16;11(3):e01157-20. doi: 10.1128/mBio.01157-20 (PMC7298714; doi:10.1128/mBio.01157-20)
Supplement: FIG S1 [file mBio.01157-20-sf001.docx]

**Supplemental Figure 1.**

*Supplemental Figure 1.* Design and testing strategy for RNA-based infection detecting multiplexed assays. From left to right, diagram shows the initial assay components included in the V1 assay, with host RNAs detected shown in black and viral RNA shown in blue. Moving right, the diagram shows that following testing, new mRNA detecting probes were added to create a V2 assay that was capable of predicting disease stage. mRNA probes added at this stage are shown in green. The final iteration involved the addition of a probe to detect MARV genomic RNA as well as additional host mRNA detecting probes (purple) to lead to a third-generation assay, the EBOV/MARV Discrimination Assay.
