# Supplementary material for: Previremic Identification of Ebola or Marburg Virus Infection Using Integrated Host-Transcriptome and Viral Genome Detection
Source: mBio. 2020 Jun 16;11(3):e01157-20. doi: 10.1128/mBio.01157-20 (PMC7298714; doi:10.1128/mBio.01157-20)
Supplement: FIG S2 [file mBio.01157-20-sf002.docx]

**Supplemental Figure 2**

Supplemental Figure 2: Pre-viremic identification of infection in MARV exposed NHPs using a NanoString assay. A) Blood samples from MARV exposed NHPs used in this study. Each black dot represents one blood sample taken at the indicated time post-infection. B) Sample correlation to expression profiles determined on the EBOV samples. Nanostring RNA level results from each sample (X-axis tic mark) were compared to pre-infection, early infection and late infection RNA accumulation profiles, and the extent of correlation to pre-infection (black) early (blue) and late (red) profiles is shown. In this analysis perfect correlation equals one. The highest correlation value would determine if the samples is classified as infected or uninfected with early and late infection both representing infected.
