# Supplementary material for: Previremic Identification of Ebola or Marburg Virus Infection Using Integrated Host-Transcriptome and Viral Genome Detection
Source: mBio. 2020 Jun 16;11(3):e01157-20. doi: 10.1128/mBio.01157-20 (PMC7298714; doi:10.1128/mBio.01157-20)
Supplement: FIG S3 [file mBio.01157-20-sf003.docx]

**Supplemental Figure 3**

Supplemental Figure 3: Host RNAs that show differential early expression in Ebola virus infected NHPs as compared to Marburg, Lassa, or Yellow Fever infected Animals. A. Each graph depicts the normalized log-fold change of one host mRNA at increasing times post-infection to EBOV (black), MARV (red), or LASV (blue). Analysis was done using data from GS64538, PRJNA222892, and PRJNA222891. Top 4 graphs represent mRNAs that showed selective upregulation in EBOV but not MARV or LASV at early times post-infection. Bottom graphs represent mRNAs that showed selective downregulation in EBOV infected NHPs at early times post-infection. B. Each graph depicts the normalized log-fold change of one host mRNA at day 3 post-infection compared to day 0 post-infection for Yellow Fever (YF), Marburg (MARV), Lassa, (LASV), and Ebola infections (EBOV).
