# Supplementary material for: Previremic Identification of Ebola or Marburg Virus Infection Using Integrated Host-Transcriptome and Viral Genome Detection
Source: mBio. 2020 Jun 16;11(3):e01157-20. doi: 10.1128/mBio.01157-20 (PMC7298714; doi:10.1128/mBio.01157-20)
Supplement: FIG S4 [file mBio.01157-20-sf004.docx]

**Supplemental Figure 4**

Supplemental Figure 4: Appearance of EBOV and MARV genome RNA in blood samples from EBOV and MARV infected NHPs using NanoString. A) Normalized probe count values for an EBOV genome-specific RNA probe. Each point represents normalized counts from an individual blood sample on the indicated day postinfection. Horizontal line indicates average value with error bars signifying standard deviation. Dashed line represents background threshold for the probe. Samples from EBOV-infected NHPs are shown in black, and samples from MARV-infected NHPs are shown in red. B) Similar graph to A) but showing results from the MARV-genome-specific RNA probe. Arrow shows the early infection timepoint (3DPI).
